# Game-based learning in undergraduate medical education: evaluation of an interdisciplinary escape room

**DOI:** 10.1186/s12909-025-07990-2

**Published:** 2025-11-15

**Authors:** Franziska Baessler, Torben P.P. Hornung, Tom Johann Schuster, Nina Hardt, Emilia Felter, Elisabeth Anzenberger, Daniela Roesch Ely, Theresa Lichtenstein, Katharina Steiner, Katja Koelkebeck, Vera Flasbeck, Philipp Spitzer, Sven Speerforck, Severin Pinilla, Elham Khatamzas, Barbara Mueller, Ali Zafar

**Affiliations:** 1 Center for Psychosocial Medicine, Department of General Psychiatry, Heidelberg University Hospital, Thibautstraße 4, 69115 Heidelberg, Germany; 2 Department of Psychiatry and Psychotherapy, Cologne University Hospital, Cologne, Germany; 3 Department of Psychiatry and Psychotherapy, Essen University Hospital LVR, Duisburg-Essen University, Essen, Germany; 4 Department of Psychiatry and Psychotherapy, Bethel Evangelical Hospital, Bielefeld University Hospital OWL, Bielefeld, Germany; 5 Department of Psychiatry, Psychotherapy and Preventive Medicine, LWL- University Hospital, Ruhr University Bochum, Bochum, Germany; 6 Department of Psychiatry and Psychotherapy, Erlangen University Hospital, Erlangen, Germany; 7 Department of Psychiatry and Psychotherapy, Leipzig University Hospital, Leipzig, Germany; 8 University Hospital of Old Age Psychiatry and Psychotherapy, Bern, Switzerland; 9 German Center for Infection Research (Partner Site), Heidelberg University Hospital, Heidelberg, Germany; 10 Department of Infectious Diseases (Virology), Heidelberg University Medical Faculty, Heidelberg, Germany

**Keywords:** Escape room, Gamified teaching, Gamification, Infectious diseases, Psychiatry, Medical education, Didactics, Game-based teaching

## Abstract

**Background:**

Educational escape rooms are an innovative pedagogical approach to encourage proactive learning among students. This study assessed the learning outcomes and student satisfaction with escape room-based teaching at a medical school in Germany.

**Methods:**

An interdisciplinary escape room was created based on learning goals from the fields of psychiatry, infectious diseases, and communication skills. The gameplay was repeated eight times and pre-post intervention questionnaires were used to assess knowledge gain of participants. Pre- and post-test scores were analyzed using t-tests and correlation analyses to examine their relationships with gender, age, and semester. Evaluation feedback was summarized with descriptive statistics (mean, SD). Three open-text questions recorded qualitative comments, which were analyzed thematically.

**Results:**

Overall, 45 students (female = 82.2%; male = 17.8%; *M*_*age*_=24.04 years, *SD*_*age*_=3.45) fulfilled inclusion criteria. The mean post-test score (*M* = 71.71, *SD* = 5.03) was significantly higher (*t* = 8.65, *p* < .001) than the mean pre-test score (*M* = 65.07, *SD* = 5.29). Female students scored higher than male students in pre-tests (*r* = .306, *p* = .041) and post-tests (*r* = .440, *p* = .002). A higher semester of study correlated with higher pre-test scores (*r* = .536, *p* < .001) and higher post-test scores (*r* = .411, *p* = .006). Students rated the teaching method as “good” (*M* = 1.58; *SD* = 0.53) and “recommendable to peers” (*M* = 1.20; SD = 0.45), “fun to play” (*M* = 1.22; SD = 0.52), and “relevant for teaching” (*M* = 1.24; SD = 0.48). Students mentioned collaborative teamwork as the best characteristic of escape room teaching and suggested comprehensive briefing and debriefing sessions for improving future sessions.

**Conclusions:**

Escape room-based teaching resulted in significant knowledge gain. Female gender was associated with higher pre- and post-test knowledge. Students at later stages of their studies had higher pre-test knowledge. The escape room was well perceived by medical students and considered an enjoyable learning environment for medicine-related topics.

**Supplementary Information:**

The online version contains supplementary material available at 10.1186/s12909-025-07990-2.

## Introduction

Medical education has increasingly transitioned from passive learning via lectures and seminars to more activating pedagogical strategies. To facilitate student-centered teaching, simulated patients, blended learning, bedside teaching, online virtual rotations and problem-based learning have become mainstays of medical curricula in recent years [1–5]. An increasingly popular teaching approach in medical education is the use of game-design elements labelled as “gamification” or “game-based learning” (GBL) [6–8]. Gamified education stems from the concept that like video games capture the attention of players and generate an intense and lasting commitment, the use of game elements in an educational context can also make teaching and learning more enjoyable and engaging [9, 10]. While the scope of GBL is extensive, gamification can be described as a process of game-thinking and game mechanics to engage users and solve problems [11, 12]. Gamification is one of the strategies of GBL, whereby game design elements are incorporated into traditionally nongame contexts like medical education [13, 14]. Recent evidence suggests that GBL and gamification provide students with an intensive learning experience with positive group dynamics, improves learning outcomes by motivating students [6, 15], increases engagement and encourages social interaction within a learning environment [6, 16].

Within the realm of GBL, the use of escape rooms in medical education is gaining traction among teachers and researchers [17]. An actual escape room is a “live-action team-based game in which players discover clues, solve puzzles, and accomplish tasks in one or more rooms in order to achieve a specific goal (usually escaping from the room) in a limited amount of time” [18]. Although teaching in an escape room may take different pedagogical approaches, the main concept is to create a scenario where students should perform several tasks either as a group or individually within a set timeframe [19]. In “medical” escape rooms, the players are supposed to complete a series of medically themed puzzles using clues within the thematic context that allows them to solve or escape the emergency situation [20]. The use of educational escape rooms has grown internationally [21] and medical escape rooms have been successfully designed and implemented for teaching students and professionals about patient safety [22], nursing [23, 24], and emergency medicine [25]. Teaching via virtual escape rooms also gained popularity during the COVID-19 pandemic when physical and social distancing measures suspended routine teaching activities at medical schools [25, 26].

Game-based medical education in Germany is still in its formative years, although consistent efforts have been made to modernize the medical curriculum by replacing the traditional learning focus from subject-based knowledge to competency-based learning, patient-centered care, interprofessional education, and scientific competencies [27]. A new version of the German national guidelines for medical education was released in 2021 to serve as the basis for revising the medical curricula for the new German medical practice regulations, which are also currently under revision and set to be approved in 2027 [28]. However, despite calls by educators to modernize and improve medical education through innovative teaching methods, medical schools have been slow to implement game-based learning, and few examples of gamified teaching methods exist.

For this study, we designed and implemented an interdisciplinary medical escape room at the Heidelberg University Medical Faculty. The activity was based on a number of learning goals specified for psychiatry, infectious diseases and communication skills in the German national guidelines or the National Competence-Based Catalogue of Learning Objectives for Undergraduate Medical Education (NKLM). The gameplay was aimed at providing students with broad interdisciplinary knowledge about psychotic disorders, one potentially deadly infectious disease and its medication, proper patient handling, diagnostics, therapy and infection prevention and control implications. To determine the efficacy and feasibility of the escape room, we assessed the learning outcomes and student feedback to explore its value as a pedagogical approach in undergraduate medical education.

## Methods

This cross-sectional study evaluated the learning outcomes of a medical escape room, potential group differences, and student satisfaction with this innovative pedagogical intervention. The study was approved by the ethics commission of the Heidelberg University Medical Faculty (S-004/2024).

The interdisciplinary escape room was created at the Heidelberg Medical Faculty, covering certain topics from psychiatry, infectious diseases, and communication skills. A 40-minute gameplay based on puzzles, tasks and clues was followed by a 30-minute debriefing session similar to other teaching methods. During the debriefing, students received feedback on their performance from three instructors and reflected back on their learning experiences. Reflective observation holds crucial importance in the learning process, allowing students to analyze and make sense of their experiences [29, 30].

**Fig. 1:**
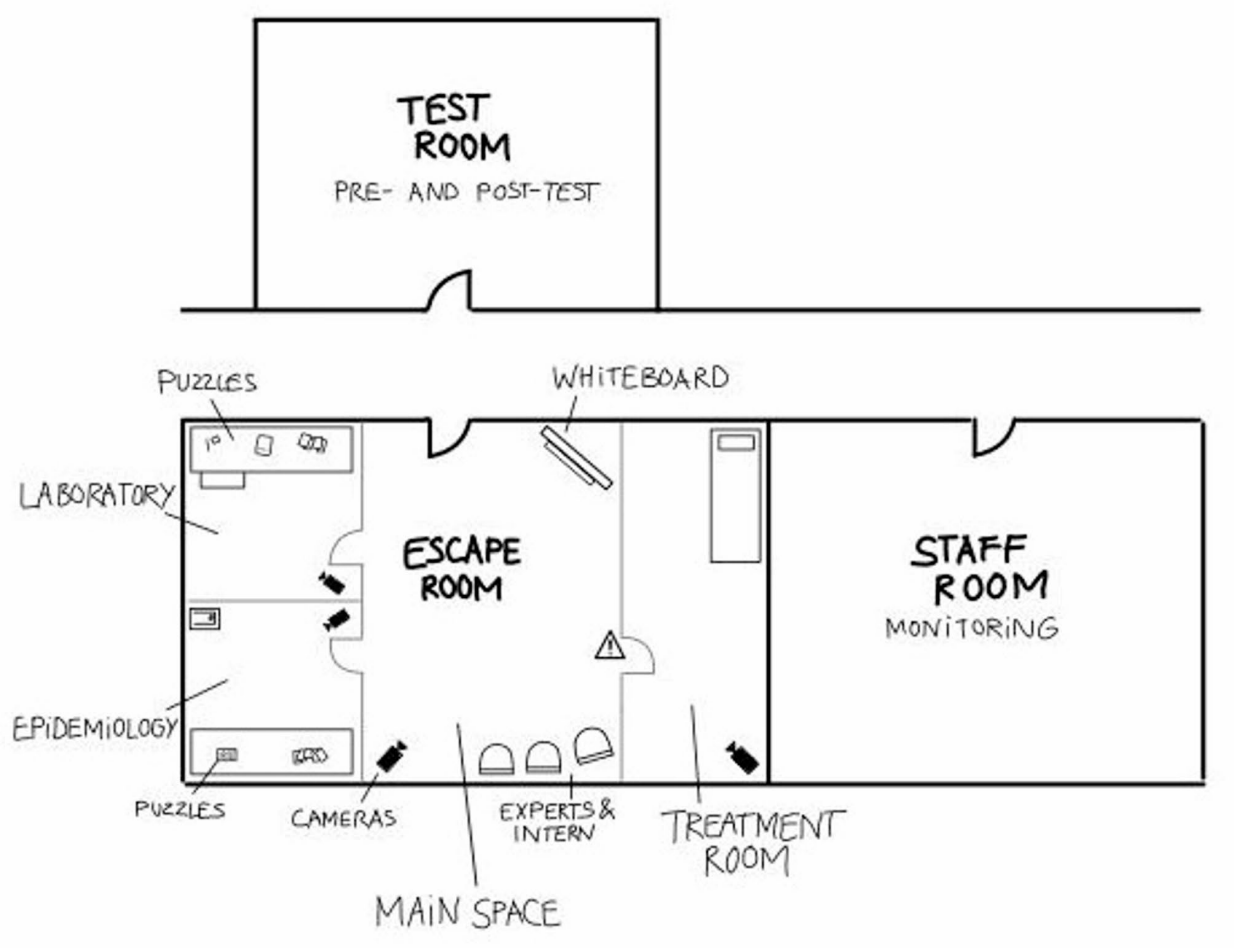
An illustration of the escape room layout and the adjacent observation deck setup. Participants arrive in the test room for an introduction and for completing the pre-test questionnaire in case they have not completed it online beforehand. After the briefing, the participants enter the adjacent escape room, where the gamemaster introduces them to a patient and the background story. The patient is taken to the treatment room for isolation and anamnesis as the 40-minute gameplay starts. A 30-minute countdown is initiated after students leave the treatment room and are free to move around and solve puzzles by finding clues in the “epidemiology” and “laboratory” rooms. The research team monitors the gameplay from the staff room for detailed feedback during the debriefing

Pre- and post-intervention tests were used to determine knowledge gains, student satisfaction and qualitative feedback on desired improvements. The escape room (Fig. 1) was repeated eight times between January and June 2024. The complete concept and design of the escape room along with the learning goals and timelines are included in the supplementary material: Appendix A provides the escape room’s general game design and learning goals. Descriptive storyline and all necessary organization details are provided in Appendix B and C, respectively.

### Recruitment

Students enrolled at the Heidelberg University Medical Faculty were invited to participate in the escape room-based teaching session. The activity was promoted using posters, social media, the official teaching platform (Moodle) and via personal contacts. Participation was voluntary and students were not graded. Students who agreed to participate in the escape room received the link to an online “pre-test questionnaire”, ideally before the planned activity. They completed it without supervision, unlike the “post-test questionnaire”, which was completed under supervision on-site, directly after the activity.

The participants provided their consent online and agreed to the data privacy statement before starting the pre-test questionnaire. The questionnaires were available on LimeSurvey as well as in paper format. Only students who participated in the escape room were eligible to participate in the study. Inclusion criteria included enrollment in medical studies, legal age of consent (≥18 years) and consent to scientific use of data. Exclusion criteria were lack of legal age (< 18 years) or lack of consent to the scientific use of data.

### Measurements

Pre-and post-test questionnaires, each consisting of 19 similar multiple-choice questions (MCQs) with five answer options, were designed specifically for this study to measure objective knowledge before and after the escape room (Appendix D). The MCQs were formulated after Delphi group discussions [31] involving three medical educators (psychiatrists), two infectious diseases experts, and two medical students. After two rounds of discussions, comments and adaptions, the MCQs were finalized with 100% consensus.

Each question was derived from a specific learning goal described in the NKLM and mainly referred to subjects taught in the clinical semesters. In Germany, medical education comprises a minimum of 6 years (12 semesters) divided into pre-clinical (semester 1–4) and clinical semesters (semester 5–12). The same knowledge-based questions were repeated in the post-test questionnaire, and 15 additional questions were added to evaluate the escape room teaching methodology. These evaluation questions were derived from instruments used in similar studies [32–34]. Each question was rated on a Likert-like scale, ranging from 1 = strongly agree to 6 = strongly disagree.

To assess the students’ subjective knowledge of the topics, one question asked them to rate themselves on a scale similar to the 6-point German school grade from 1 = very good to 6 = unsatisfactory.

At the end of the questionnaire, two open-ended questions were used to obtain qualitative information on “best aspects” and “improvements” in the escape room. The questions were: “What did you like best about this escape room?” and “What changes would you suggest to improve the escape room?”. Another open-text question asked the participants about other suitable medical subjects/topics for escape room-based teaching.

### Data analysis

We used a mixed-methods approach to evaluate students’ learning outcomes, satisfaction with teaching and the overall potential of the teaching methodology.

In the pre-post knowledge assessment questionnaires, each of the 19 MCQs comprised five answers of one point each with multiple correct options, corresponding to a total score of 95 points. Thereby, students scored one point each for choosing every correct answer and one point each for not choosing the incorrect answers. This scoring methodology has been used for capturing partial knowledge about the topic and for reducing the possibility of guessing by students [35, 36]. Evaluating each response as a true/false decision and scoring points for both correct selections and correct omissions is an effective scoring approach for “select all that apply” questions [37]. For an overall grade, the individual score was calculated and compared with the German school grading scale to create cut-off points, i.e. Grade 1 (≥86 points out of 95), 2 (76–85 points), 3 (67–84 points), 4 (57–66 points), 5 (46–57 points) and 6 (≤ 47 points out of 95) [38]. Therefore, the difference between scores of pre- and post-test MCQs was considered as the knowledge gain, which was calculated by mean and standard deviations.

Paired T-tests were used to compare the mean pre-test and post-test scores. Effect sizes were calculated with Cohen’s d [39]. Two-sided Pearson correlational analysis provided an understanding of potential influencing factors on the pre-test and post-test scores as well as on the learning outcome. Correlations were calculated between demographic variables (such as gender, age, semester of study). The results were considered significant at *p* <.05. Given the exploratory nature of the subgroup analyses, no formal correction for multiple comparisons was applied. However, we acknowledge the increased risk of Type I error due to the number of tests (*n* = 11). For transparency, we report which correlations remain significant when applying a Bonferroni-adjusted threshold of *p* <.0045.

For the evaluation of the teaching method, means and standard deviations were calculated for each of the 15 ordinal-scaled variables, offering an overview of satisfaction among participants. Quantitative data were analyzed using SPSS (Statistical Package for Social Sciences, IBM, Version 27.0).

Answers to three open-text questions were analyzed using qualitative content analysis [40]. The coding process was carried out by two authors (FB and EF) via inductive category formation from repetitive sequences to identify thematic categories. All responses were evaluated verbatim by both authors independently to generate a coding system. Inconsistencies were discussed after the first round of coding to establish intercoder agreement. The codes were refined after second round and final categories were agreed upon after consensus between the two coders. The categories and example quotes were translated into English.

## Results

Overall, 45 medical students (female = 82.2%; male = 17.8%) fulfilled the inclusion criteria for participation. The students were aged between 20 and 35 years (*M* = 24.04, *SD* = 3.45) and enrolled between the 2nd and 16th semesters, with seven students in pre-clinical semesters (1st-4th semester) and 38 students (one missing value) enrolled in clinical semesters (5th semester onwards).

Two participants were excluded from data analysis: one was not enrolled in medical school, and another was identified as an extreme outlier based on the difference between pre-test and post-test scores (*diff*_*post−pre*_=−15). The participant’s score differed by more than three SD from sample mean (*Z*_*−15*_=−3.52 with *SD* = 6.01 and *M* = 6.17). Given that *IQR* = 8 (*Q1* = 3; *Q3* = 11), the lower outlier threshold was calculated as *Q1*−1.5*IQR = 3-1.5*8=−9. Any value below − 9 was considered an outlier. No other cases met these criteria on both ends of the spectrum.

### Knowledge assessment

The overall mean pre-test score was 65.07 (*SD* = 5.29) out of 95, while the mean post-test score was 71.71 (*SD* = 5.03), which was a significant improvement (*t* = 8.65, *p* <.001) with a mean knowledge gain of 6.64 points (SD = 6.01, 95% CI [4.84, 8.44]) and a large effect size (Cohen’s d = 1.29, 95% CI [0.89, 1.69]). A two-sided Pearson correlation showed a significant negative correlation between the pre-test score and knowledge gain (*r*=-.536, *p* <.001, 95% CI [−0.717, − 0.288]), indicating that higher pre-test scores were associated with lower knowledge improvement. Four students scored lower in the post-test than their pre-test.

Female participants scored higher than male students in the pre-test (*r* =.306; *p* =.041, 95% CI [0.014, 0.550]) as well as in the post-test (*r* =.4404, *p* =.0024, 95% CI [0.169, 0.650]). The mean pre-test score for male students was 61.63 (*SD* = 6.21) and for females 65.81 (*SD* = 4.85). In the post-test, the mean score for male participants was 67.00 (*SD* = 4.14) and for female students 72.73 (*SD* = 4.65).

The students’ semester level was positively correlated with higher pre-test (*r* =.536, *p* <.001, 95% CI [0.288, 0.717]) and post-test (*r* =.411, *p* =.006, 95% CI [0.134, 0.629]) scores, but not with greater knowledge gain from the intervention (*r*=-.153, *p* =.322, 95% CI [−0.427, 0.147]). Age was also significantly correlated with higher pre-test scores (*r* =.346, *p* =.020, 95% CI [0.058, 0.581]), but not higher post-test scores (*r* =.167, *p* =.273, 95% CI [−0.133, 0.439]) or knowledge gain (*r*=-.192, *p* =.206, 95% CI [−0.460, 0.108]).

**Fig. 2:**
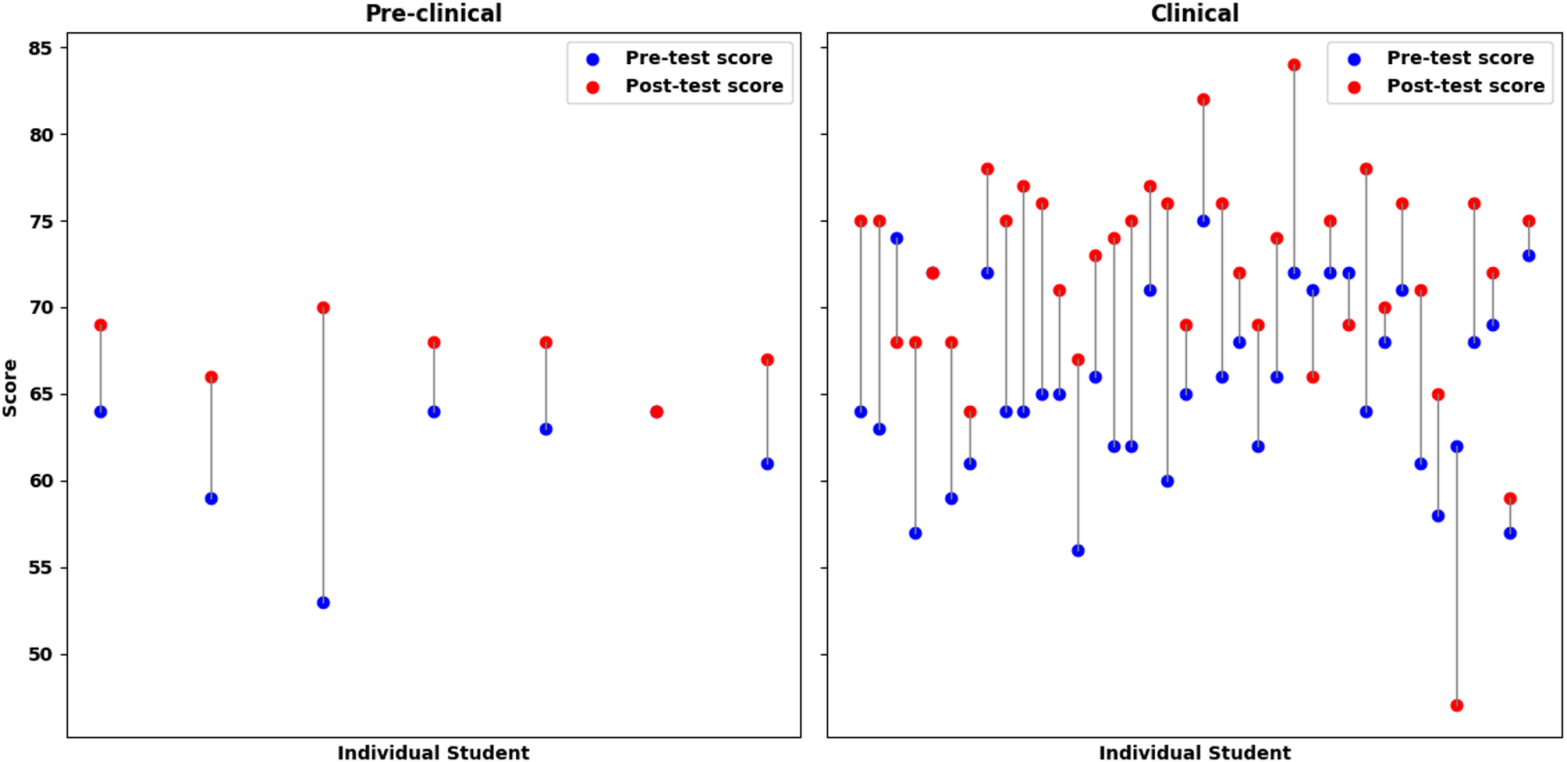
The differences in individual pre-post test scores (out of 95), illustrating the overall knowledge gain for all participants

These correlations remained statistically significant after applying Bonferroni correction for multiple comparisons. Other reported associations should be interpreted as exploratory findings. Figure 2 shows the individual pre-post test scores (out of 95), illustrating the overall knowledge gain for all participants.

### Self-assessment and teaching evaluation

For the self-assessment of subjective knowledge, the students’ mean grade was 4.36 (SD = 1.04) in the pre-test and 3.33 (SD = 1.00) in the post-test. The participants’ actual mean grade improved from 3.71 (SD = 0.55) in the pre-test to 2.89 (SD = 0.61) in the post-test.

Similarly, their self-assessment accuracy also improved post-intervention. In the pre-test, 12.50% (1 out of 8) of male students and 32.43% (12 out of 37) of female students accurately self-assessed their grades. In the post-test, 50% (4 out of 8) of male students and 40.54% (15 out of 37) of female students correctly estimated their grades.

Overall, the escape room experience was rated with a German school grade of 1.60 (*SD = 0.62*). Students’ feedback on the evaluation of the escape room, based on a combined scale of 15 items, was rated as “good” (*M* = 1.58; *SD* = 0.53). The highest evaluation ratings were given for “recommending the escape room to other students” (*M* = 1.20, *SD* = 0.46), “having fun” (*M* = 1.22, *SD* = 0.52), “relevant for future physicians” (*M* = 1.24, *SD* = 0.48), and “positive learning atmosphere” (*M* = 1.44, *SD* = 0.62).

Table 1 shows the scores for each variable on a 6-point scale from 1 (very good/strongly agree) to 6 (very bad/strongly disagree). 

**Table 1:** The ratings for each evaluation variable used to assess the escape room-based teaching (The questions have been translated from German into English and edited for further clarity)

Questions	Min.	Max.	Mean	SD
Please rate the course overall.	1	4	1.60	0.618
I had fun during the escape room.	1	3	1.22	0.517
The teaching format was suitable to increase my knowledge.	1	5	2.04	1.021
The teaching session increased my interest in the topics covered.	1	5	1.64	0.857
The teaching format motivates you to deal with the topics covered before or after the teaching unit/encourages self-study.	1	5	1.60	0.963
The learning atmosphere during the teaching session was positive.	1	3	1.44	0.624
The teaching format is suitable for learning content in the long term.	1	4	1.49	0.815
The teaching session helped me to identify my weaknesses.	1	5	1.58	0.866
The teaching format encouraged the active use of communication skills.	1	5	1.80	0.919
The teaching format promoted teamwork (collaboration skills).	1	5	1.62	1.007
The teaching format encouraged the use of leadership skills.	1	5	2.24	1.026
The teaching format encouraged active participation.	1	4	1.49	0.759
The topics covered are relevant for future physicians.	1	3	1.24	0.484
The escape room lesson was challenging and made me think.	1	5	1.51	0.869
I would recommend this course to other students.	1	3	1.20	0.457

### Best teaching aspects

All participants (*n* = 45) provided answers to the open-text questions. The length of the comments ranged from two to 42 words.

On the question of “what did you like best about this escape room?”, 16 students mentioned collaborative teamwork with comments such as “*the interactivity was fantastic!*”, “*teamwork was essential*”, “*collaboration with others*” and “*working together as a team*”. Comparing the escape room with traditional teacher-centered sessions, one student wrote:

*“Exchanging ideas with other students outside the format of ‘everyone sitting at a table and the lecturer/professor asking questions*,*’ being less inhibited about saying something wrong*,* and solving the case together [were best aspects].”*

Teaching using a clinical case and a broad scope of content and competences (mentioned 14 times) were appreciated such as:

… I liked the many skills that were integrated. For example, hand disinfection, repositioning patients, communication, and then, of course, the technical information.

[…] the teaching content seemed much more relevant and approachable through the clinical context.

In principle, I can imagine using this format for any subject in the context of such a playful presentation of patient cases. For example, as a replacement/supplement to POL.

‘Problem-solving skills’, ‘using riddles’ and ‘having fun’ (all 13x) were stated as best aspects of the escape room, for example “*combining the individual small puzzles into a complete picture*”, “*interactive puzzle solving*” and “*tracing the infection route*”. About the gameplay, one student wrote:

It was never boring, and there was always a clear thread guiding you through the game.

The opportunity for students to become active and be creative (8x), the inclusion of digital tools, simulation cases, and simulated phone calls (all 6x) were among the other most valued teaching aspects, for example:

[…} a taste of what everyday life at the clinic might be like.

Interaction with actors/external people is a very good idea.

*“…highlights were the surprise visit from the ‘hygienist’ and the ‘psychiatric patient’.”*

Regarding suggestions for improvement, 10 participants indicated a preference for more comprehensive briefing sessions and clearer instructions. Students also suggested small groups of up to a maximum of six players (mentioned 9 times) and expressed a desire for more playing time (6x).

On the question about the suitability of medical subjects for future escape rooms, 13 students believed all medical subjects could be taught using this format. Subjects perceived to be most suitable were infectious diseases (mentioned 13 times), emergency medicine (12x), internal medicine (9x), orthopedics (4x) and trauma surgery (4x).

## Discussion

This study evaluated an innovative escape room concept to determine the learning effects of gamified teaching and its effectiveness as a teaching methodology. To the best of our knowledge, this was the first time that an interdisciplinary escape room was tested as a pedagogical approach at a medical school in Germany. Our results showed both an overall improvement in academic performance and a notable increase in self-assessment accuracy. The participants also evaluated the teaching methodology positively and provided constructive feedback for improving future gameplay.

Our findings showed a high knowledge gain after the escape room teaching sessions, recorded by the considerably higher post-intervention scores of students and a large effect size. The mean post-test score of participants increased by six points despite four students scoring lower in the post-tests than their pre-tests. It is worth noting that many students had completed the online pre-test questionnaire unsupervised before coming to the escape room, whereas the study staff supervised the post-test questionnaire immediately after the gameplay. While it introduced testing condition bias, the mean post-test scores increased even though direct supervision reduced the chance of participants looking for correct answers on the internet.

However, improved learning outcomes among students have been reported previously with escape room-based teaching in other medical specializations [14–17]. Studies show that game-based learning can provide medical students an intensive learning experience, promoting better learning outcomes than passive teaching formats [34, 41, 42]. The effectiveness of gamified learning in promoting knowledge retention and skill acquisition is supported by Kolb’s experiential learning cycle [29], underpinning the active, reflective, and iterative nature of gameplay that enables students to apply and refine knowledge in context. When implemented in teams, game-based learning also aligns with team-based learning [43], promoting accountability, collaboration, and deeper engagement with content. Furthermore, cognitive load theory highlights how games can reduce extraneous cognitive load through structured environments and increase germane load by encouraging meaningful problem-solving [44]. These frameworks provide a strong pedagogical rationale for integrating game-based approaches in medical education.

Age and semester of study significantly influenced pre-test scores in our study, with older students in higher semesters scoring relatively higher than younger students in earlier semesters. These findings are evident of prior exposure to subject knowledge of medical students as they progress in their studies. As students advance through semesters, they demonstrate significant improvements in understanding and application of medical concepts, indicating a clear trajectory of knowledge improvement [45]. A longitudinal study reported significant increases in perceived competence across various domains, including medical knowledge and patient care, as students progressed through their years [46]. Medical students at Heidelberg University are taught the basics of infectious diseases in their 5th semester while psychiatry is taught between 8th and 10th semesters. It is worth noting, however, that age and semester did not affect post-test scores or knowledge gain, suggesting that the escape room resulted in higher learning outcomes among younger students enrolled in earlier semesters. The correlation tests confirmed that students who scored higher in pre-test, were older and in higher semesters, and did not learn as much as students who scored lower in pre-test, who were younger and in earlier semesters. This finding suggests that escape room teaching may be particularly beneficial for younger students in earlier semesters or those with lower threshold of knowledge about the topics. However, this score only measured the difference in theoretical knowledge gained, not the other important skills characteristic of gamified teaching such as interprofessional learning, working under time pressure, or communication skills. The high pre-test scores may also be due to selection bias as only students interested in the topic with prior subject exposure might have participated in the escape room. Nevertheless, this observation may be notable for convincing medical schools and lecturers to offer cross-semester teaching. While lecturers may think it is necessary to provide theoretical background before performing practical exercises, these findings suggest that more student-centered learning, similar to flipped classrooms [47–49], might be feasible.

Our findings also suggested that female gender was associated with higher pre-test and post-test scores, suggesting relatively higher subject knowledge among female students than their male counterparts. Previous research suggests female students tend to demonstrate relatively higher subject knowledge across various health science disciplines [50, 51], although the extent of this advantage may vary depending on the specific field and assessment method. While about two-thirds of students in German medical schools identify as females [52], our sample population was overly representative of female medical students (82%), restricting us from drawing general conclusions on gender differences. Combined with convenience sampling from a single institution, the high representation of female students considerably affects the generalizability of these outcomes. Future studies could determine gender differences in gamified learning via stratified sampling to enable robust subgroup analyses.

Before the escape room, about one-third of students accurately evaluated their knowledge, increasing to nearly half post-intervention, indicating improved self-awareness. Male students tended to slightly underestimate their knowledge compared to females, contrasting with previous findings [53]. While medical students generally struggle with self-assessment [54], prior studies suggest females tend to underestimate their performance more than males [55]. However, the low number of male participants in this study limits further conclusions. It is important to note that several subgroup correlations did not remain statistically significant after correcting for multiple comparisons. Given the exploratory nature of the analyses, these findings should be interpreted with caution. Future studies with larger, more homogenous samples and confirmatory designs are needed to test these associations.

Overall, our participants rated the escape room as “good” on the German school grading system. Among the best aspects of the teaching format was related to having fun during the educational session. This is supported by earlier studies that suggest aspects of fun-based learning and solving problems together as a team are essential pedagogical tools for improving learning outcomes [6, 15, 16]. A well-designed immersive experience within a safe learning context, balanced between competition and fun can encourage participation, maintain interest and motivate students by increasing their social engagement within a learning environment [16, 56]. Also among the best characteristics, teamwork and solving riddles emerged as the most appreciated aspects of the escape room learning experience. It is worth noting that knowledge scores alone did not capture the gain in soft skills such as communication and teamwork, which recent studies have found to be important learning outcomes of GBL [6, 15, 16]. We recommend future studies on developing an evaluation framework on acquisition of non-cognitive skills in gamified medical education.

The students also appreciated the use of a clinical case during the escape room, which draws similarities with other didactic methods of advanced skills lab or a patient simulation. From a learning perspective, the students rated the escape room to be “suitable for memorizing learning content in the long term”, for “encouraging active participation, “relevant for topics for future physicians”, and a “challenge that made me think”. These aspects also align with previous research which shows that “learning by doing” during escape rooms leads to a higher knowledge gain and recall capacities in different medical fields [33, 56–58]. The impact of escape room-based teaching has been reported earlier on enhanced students’ engagement and motivation [59, 60], clinical judgment [61] and critical thinking skills [62]. Despite the positive feedback, however, it should be noted that escape rooms are still a new concept for medical education in Germany and further research is necessary to measure their long-term learning effects. Future studies could also explore how this pedagogical method affects team dynamics and whether potential positive outcomes translate to clinical contexts by developing specific evaluation frameworks.

## Conclusions

Our results showed a significant knowledge improvement among medical students after participating in the escape room. The participants evaluated the teaching methodology positively and provided constructive feedback for improving future sessions. However, the relatively small sample size and heterogeneity in study semesters make it difficult to generalize our findings. Moreover, participants from a single medical institution share a relatively homogenous educational environment, thereby influencing both the reception and learning outcomes of the intervention. The results may not be replicable in institutions with different curricular structures or student support systems. Therefore, we only identified correlations but did not draw causal conclusions. This may be addressed in the future with larger studies, systematically comparing different phases of the medical curriculum or supported by control groups. Nevertheless, medical schools could explore the possibility of introducing gamified education in the form of escape rooms similar to other patient simulation-based instructional strategies.

When systematically implemented, escape rooms could provide an immersive interprofessional educational experience for students while enabling teachers to observe gaps in knowledge. Since the design and implementation of an escape room are time consuming and resource-intensive, it cannot fully replace standard teaching methods. However, it complements such methods by providing an engaging and collaborative learning experience, which may be particularly beneficial for students with lower initial knowledge levels. Furthermore, it trains essential capabilities beyond fact-based knowledge, such as communicating, collaborative teamwork, and making decisions under time pressure. Successfully developed and tested storyboards may be shared and implemented by other medical faculties.

## Supplementary Information

Supplementary Material 1.

Supplementary Material 2.

Supplementary Material 3.

Supplementary Material 4.

## Data Availability

The dataset used and analyzed for this study can be provided by the corresponding author on reasonable request.
