# Supplementary material for: Game-based learning in undergraduate medical education: evaluation of an interdisciplinary escape room
Source: BMC Med Educ. 2025 Nov 15;25:1606. doi: 10.1186/s12909-025-07990-2 (PMC12619439; doi:10.1186/s12909-025-07990-2)
Supplement: Supplementary file 1 — Supplementary Material 1. [file 12909_2025_7990_MOESM1_ESM.docx]

**Appendix C**

**Toolkit for Creating an Educational Escape Room**

## Preparation Phase

### 1. Form a Team

Gather a diverse group of individuals with skills in medicine (respectively specialists in the field of your learning goals), education (if possible, with experience in game-based teaching or escape room design), and in event planning. Assign roles according to competences such as team manager, content creator, logistics coordinator, and technical support.

### 2. Create a Storyline and Define Learning Goals

Select a clinical case that aligns with your educational objectives. Develop clear learning goals that the escape room will address. Craft a compelling storyline that integrates these learning objectives in a fun and engaging manner.

### 3. Plan the Concomitant Study (Optional)

If you choose to conduct a study alongside the escape room activity, outline the study objectives, methodology, and data collection processes. Determine what metrics will be measured, such as participant performance, engagement levels, or learning outcomes. Ensure ethical considerations and approvals are addressed and prepare the necessary documentation for conducting the study.

### 4. Write a Screenplay

Detail the flow of the game, including the sequence of events, timing, and roles for each participant. Include specific instructions for each simulation personnel and the team overseeing the game. (For further guidance, please refer to the section on Storyline.). It will be useful if you decide to write a publication.

### 5. Recruit and Train Simulation Personnel

Recruit individuals who can act as simulation persons (e.g. patients or healthcare staff) during the game, in person, online or by phone, as required. They must be briefed about their roles in advance and, if possible, trained before the gameplay.

### 6. Invite Experts to Supervise

## It is recommended that experts from the field of the learning goals are brought in to oversee the process, provide feedback, and ensure that the educational goals are met.

### 7. Invite Students

Schedule small rounds of around 6 students per session. Plan multiple rounds throughout the day to maximize use of resources. Double check to avoid late drop-outs. It might be wise to invite more students depending on the likelihood some will not be able to participate spontaneously or to have a waiting-list.

### 8. Book Several Rooms

- Escape Room: The main area where the game will take place.
- Team Room: For the organizing team and simulation persons to stay, coordinate and manage the event. If possible organize food and drinks since it might be a long day.
- Lounge: A space for participants to wait, store belongings, and potentially engage in pre- and post-tests, if the game is study-based.
- Storage room: A secure space where equipment can be stored on a long-term basis. This eliminates the need to transport all of the equipment each time the game is played.

*An example of the rooms and setup used in the escape room discussed in this publication.*

### 9. Prepare Requisite Materials

Collect all materials needed for the escape room puzzles and clinical scenarios. Ensure all props, documents, and tools are ready and functional.

### 10. Install Cameras and Microphones (Optional)

Set up audio-visual equipment for live streaming or recording for evaluation purposes. It is beneficial to be able to observe the progress of the escape room from another room. It is important to note that the input of experts in the escape room is highly valuable. However, students may feel more comfortable if they can observe from a nearby room. In some locations, rooms are equipped with a mirrored window, which is a valuable resource.

### 11. Ensure Data Protection

Ensure that all participants provide informed consent if you plan to conduct a study involving the escape room. This is particularly crucial if you intend to record video or audio during the activity. Clearly communicate the purpose of the recordings, how the data will be used, and how it will be protected to maintain privacy and confidentiality.

# Conduction Phase

### 1. Timing the Team

Ensure all team members know their schedules, tasks and responsibilities. Use a detailed timeline to manage the flow of activities.

### 2. Setup and Final Checks

Set up the escape room according to the planned design, arranging all props, puzzles, and clues. Verify the functionality of all elements and address any issues. Test locks, mechanisms, and technology components.

### 3. Conduction

Guide participants through the escape room, offering hints as needed to help them progress with the puzzles. Maintain effective communication among team members to promptly address any unforeseen events or issues that arise during the game.

### 4. Debriefing

Conduct a debriefing session after each game to discuss the learning objectives and possible improvements. Focus on both the educational outcomes and the gameplay experience.

### 5. Pre-Post Test (Optional)

# Administer pre- and post-tests to assess the knowledge and skills gained from the escape room experience. Compare results to evaluate learning progress. Send an online version of the pre-test to participants several days in advance to save time on the day of the escape room. Consider conducting a long-term post-test to determine lasting learning effects.

# Tips and Tricks

### 1. Secure Material Storage

Use a lockable room to store all materials and equipment when not in use to prevent loss or tampering.

### 2. Efficiency through Repetition

Conduct several sessions in a row to make the most of your setup and resources for the day.

### 3. Feedback by Undistracted Supervisors

Ensure supervisors are focused on observing and providing constructive feedback without other distractions. It would be beneficial to implement a checklist system, to enable supervisors to provide valid feedback easily.

### 4. Engage Medical Students

Involve medical students in the preparation phase to ensure the content is relevant and engaging from a student perspective. Particularly final year students or students, who just passed the related subjects, are familiar with the learning goals and the perspective of a medical student.

### 5. Rehearse the Escape Room

Start rehearsing the escape room from an early stage on. This helps to identify potential problems in advance and allows to refine the design. During rehearsals, also decide the context of the escape room: whether it will be used for summative or formative assessment, and if it will be part of elective or non-elective studies. Early rehearsal ensures a smoother execution and aligns the activity with your educational goals.
