# Supplementary material for: Game-based learning in undergraduate medical education: evaluation of an interdisciplinary escape room
Source: BMC Med Educ. 2025 Nov 15;25:1606. doi: 10.1186/s12909-025-07990-2 (PMC12619439; doi:10.1186/s12909-025-07990-2)
Supplement: Supplementary file 2 — Supplementary Material 2. [file 12909_2025_7990_MOESM2_ESM.docx]

**Appendix A**

**Script of the Gameplay**

| **Time (min.)** | **Scene** | **Description** | **Tasks/Learning Goals** |
| --- | --- | --- | --- |
| 0-15 | Briefing | - general information for the students: act as if they were real physicians, always do things like they would do them for real, time pressure, if someone enters the room, freeze and wait for further information, ignore experts  - students enter the Escape Room  - Briefing inside: intern explains, which spaces are "outside" and "inside" the game, show room |  |
| 15-20 | Prologue | - Dr. House (game master) enters  - Dr. House explains tasks 1)-3) & assigns students the task of taking medical history of the patient in treatment room  - Dr. House leaves | Tasks for students:  1) which pathogen causes the disease? 2) how can it be treated?  3) how can the transmission be stopped? |
| 20-25 | Anamnesis | - students must find the right isolation clothing (1) and dress correctly for isolation duty  - students take medical history of the patient Mrs. Keller (2)(3)  - students should avoid a handshake with the patient | Learning goal 1: choose correct isolation clothing for situation  Learning goal 2: effective patient physician communication  Learning goal 3: anamnesis focusing on infectiology |
| 25-30 | Wheelchair Transfer | - Mrs. Keller faints  - Dr. House comes in and gets a wheelchair  - transfer of patient from bench to wheelchair (4)  - Mrs. Keller and Dr. House leave  - task for students: solve the quest within 30 minutes | Learning goal 4: know how to correctly do a wheelchair transfer |
| 30-35 | Hygiene control | - students leave treatment room and disinfect their hands (5)  - hygiene officer enters with black light lamp  - control of hand disinfection quality at students  - hygiene officer leaves | Learning goal 5: be able to correctly disinfect one´s hands |
| 35-65 | Main phase | *(note: in this phase, dramaturgical and strategical elements like phone calls are flexible, crew and actors try to react to the progress of the individual groups)*  - intern starts countdown, students enter subspaces | 30-minute-countdown for completing the tasks 1-3 starts |
|  |  | laboratory  - clues: list of notifiable diseases (6), note pad with phone number, medication packages, rat in a box, apple juice as source of contamination (7), password for tablet (8), video of Mad (9)  - phone calls from outside | Learning goal 6: remember notifiable diseases  Learning goal 7: know the possible transmission pathways of certain infectious pathogens  Learning goal 8: be able to identify an infectious disease  Learning goal 9: know vulnerable groups for certain infections |
|  |  | epidemiology  - riddles: microscope picture (10), antibiotic medicine packages (11), liquor findings (12), safe with combination lock with patient letter of Mad inside (15)  - phone call: right dose of paracetamol (14) | Learning goal 10: be able to identify an infectious pathogen based on clinical data, microscopic image of pathogen  Learning goal 11: know correct antibiotic therapy during pregnancy  Learning goal 12: be able to interpret a liquor status  Learning goal 14: know correct pain therapy  Learning goal 15: know about the diagnosis and psychopathology of a personality disorder |
|  |  | main space:  - help from intern, students collect information on whiteboard, - students may search purse of Mrs. Keller, maternity log inside (13) | Learning goal 13: be able to correctly identify a patient |
| 65-75 | Showdown | - intern gathers students in main space, collects information on whiteboard  - Dr. House enters, asks for findings  - students present results they found (16)  - Dr. House is very thankful, leaves to cure patients  - Mad enters, asks students what they are doing in his laboratory  - students calm down Mad  - psychiatry nurse enters, finds Mad, asks if they can go, asks for Mad´s medication  - students give Mad the right medication (17) and admit Mad to psychiatry (18)  - Mad and psychiatry nurse leave  - Rat must be contained for outbreak control, because potentially infected | countdown ends  Learning goal 16: be able to do a correct clinical handover  Learning goal 17: know how to pharmacologically treat a psychotic disorder  Learning goal 18: know about medical coercive measures |
| 75-80 | End | - intern announces a time leap of 2 months  - Mrs. Keller enters, thanks students and gives a present to every student |  |
| 80-110 | Debriefing & Discussion | introduction round  - everybody (crew from outside, experts, intern, actors, students): form a circle of chairs  - everybody: introduce themselves  disclosure and discussion  - game master, crew: disclose puzzles and riddles, explain story, show wheelchair transfer  - game master, experts: explain learning goals  - students: questions and comments (19)  final round  - short feedback of everyone | Learning goal 19: be able to efficiently communicate in an interdisciplinary team |
| 110-130 | Post-Test | - post-test for evaluation of short-term learning progress |  |

**Copyright Disclaimer: If you want to replicate the script, please obtain written confirmation from the corresponding author**
