# Supplementary material for: Game-based learning in undergraduate medical education: evaluation of an interdisciplinary escape room
Source: BMC Med Educ. 2025 Nov 15;25:1606. doi: 10.1186/s12909-025-07990-2 (PMC12619439; doi:10.1186/s12909-025-07990-2)
Supplement: Supplementary file 3 — Supplementary Material 3. [file 12909_2025_7990_MOESM3_ESM.docx]

**Appendix B**

**Escape Room Story**

### Minutes 00-15: Briefing

After a short introduction to the rules, the students enter the Escape Room, which is divided in a main space and three subspaces named "Epidemiology" and "Laboratory" as well as an "Examination Room". They are welcomed by a co-game master introduced as an intern, who explains the structure of the Escape Room. The “Intern” stays in the room during the game, prepared to intervene with advice if necessary. Two experts sit on the side as silent observers who should be ignored during the game.

### Minutes 15-20: Prologue

Dr. Hope House (Co-Game-Master; tired, overwhelmed, seeking help, distracted, worried, erratic) enters the room, welcomes the students and is happy that they are now there to help. She nervously explains that there appears to be an outbreak of an unknown disease (two people have died, two others are sick). She now has another patient, Ms. Keller, with similar symptoms. She points to the whiteboard in the main space where 5 photos of patients labeled with their birth dates are displayed, two persons marked as dead. Dr. House has already tried to get an overview and points to the whiteboard with the questions:

1) What is the cause of this disease?

2) How can Mrs. Keller be treated?

3) How can we prevent further spread of the outbreak?

Unfortunately, Dr. House needs to look after other patients immediately, so she asks the participants to take a short medical history of Ms. Keller, who is waiting in the treatment room right now. Then she rushes off.

### Minutes 20-35: Anamnesis and hygiene control

The students are asked to enter the treatment room, but there is a sign with "isolation duty" at the door. The first task is to dress up with correct isolation gear first. Hand disinfectant, gowns, masks and gloves are partially provided at the door, partially hidden somewhere in the main space. Participants are asked to hurry up by the intern, since the patient is in a bad state. Inside the examination room, they meet Ms. Keller, a 20-30-year-old woman, sitting on a medical recliner. Ms. Keller greets the participants, extending her hand for a handshake. She reports about having had fever for four days, accompanied by discomfort, muscle pain, diarrhea and vomiting and is getting worse. She eventually mentions that she is four months pregnant. When she is asked whether she attended any gatherings or events recently, she mentions that she attended the graduation ceremony of her husband five days ago. Mrs. Keller starts feeling dizzy and almost faints. At this moment, Doctor House enters the treatment room (not dressed in isolation clothing). Shocked about the state of her patient, she gets a wheelchair immediately. The students need to transfer the patient from the medical recliner in the wheelchair. Doctor House takes her to the emergency room. She asks the students to answer the questions on the whiteboard as fast as possible to help her patients and prevent further transmission. While exiting the room, the patient mentions her handbag, which stays with the students.

In the main space, a timer of 30 minutes is started by the intern. The students exit the treatment room and disinfect their hands. Instantly, a “hygiene officer” appears and announces a random control. She uses a UV lamp to check the hands of all students. She leaves after giving feedback regarding the completeness of disinfection to each student. Students are assigned to two teams, who will work in “Epidemiology” and “Laboratory” to get information and share their findings in the main space until Dr. House will return.

### Minutes 35-65: Main game phase

**Main space**: In this room, the students can share their knowledge between groups and summarize their findings on the whiteboard. The intern is available for hints. The handbag of Mrs. Keller can be searched by the students.

It contains the following clues:

- A maternity log, stating Ms. Kellers birth date, blood group and vaccination status
- A bottle with liquid, labeled ‘Apple juice for Graduation ceremony, homemade’
- An invitation card for a graduation ceremony that took place xx days ago. It carries the remark: “There will be a big buffet – food or drink donations are welcome. Remember to bring your own boxes to take away leftovers.”

**Laboratory**: In the laboratory, students find a locked tablet with a note "password: 10 letters, notifiable infectious disease. Tablet will irreversibly lock after three wrong entries", a notepad and pencil, a telephone, some medical textbooks, a list of notifiable reportable infectious diseases and a few medication packages (antidepressants, antipsychotics). The following clues can be uncovered in this room:

- A photo from a party is pinned to the wall; the patients whose photos are displayed on the whiteboard are hidden within the crowd.
- The notepad has impressions from a note that was written on the previous sheet. Using a pencil to make the text visible, the students can uncover "please call" and a telephone number. If the number is called, the call is answered by a “zoo keeper” who pretends to have a language barrier and is not giving much information. He repeatedly states that he can’t take care of the rat and hangs up.
- After some minutes, there are squeaking noises. The sound comes from a box hidden under the table labelled with "Fridolin", containing a rat (toy animal, considered alive) next to a bottle labeled ‘Apple juice for graduation ceremony, homemade’.
- The phone rings, the caller is a psychiatric nurse. The nurse states to have called the laboratory, since this is the workplace of one of his/her patients, Mad Berger. The nurse asks the students if they know the patient and warns them about him being a potential danger to himself and others.
- The password that unlocks the tablet (“Listeriose”) can only be uncovered with information from the “Epidemiology team”, so sharing information between groups during the game is essential.
- The tablet contains photos of Mad and a woman, apparently Ms. Keller, lovingly hugging, and a video. In this video, Mad filmed himself while walking away from the zoo. His rat Fridolin is sitting on his shoulder. Mad explains angrily how he just got rejected from depositing his rat for custody. He threatens to apply ‘his apple juice’ to the zoo animals. He discloses that he is desperately jealous of his ex-girlfriend (Mrs. Keller) and her new husband, his old lab mate. He feels betrayed and forged his revenge plan. He poisoned apple juice with bacteria and brought it to the graduation ceremony of his lab mate. His aim was to infect the unborn child, assuming that pregnant Mrs. Keller would want to drink something without alcohol.

**Epidemiology**: In the epidemiology room, a microscope image with bacteria, medical textbooks and a telephone, some patient files labelled with "suspected cases" and packages containing different antibiotics can be found. The room contains the following clues:

- A microscopic image of gram-positive rod-shaped bacteria (Listeria)
- A safe with a number combination lock hidden in a corner
- Files from patients whose clinical and laboratory data indicate Listeriosis. Among those is a file of a patient named XXX Keller.

The phone rings and a nurse from the emergency ward urgently requests the blood type of Ms. Keller. Information on the blood type of XXX Keller can be found in the respective patient file and in the maternity log, but the information does not match. The clue is the birth date on the patient file, which indicates that this patient is only 2 years old and thus not identical with the Ms. Keller that is currently treated. Thus, the blood type listed in the maternity file is the correct answer.

Another call from a nurse asks for the maximum daily mg dose of paracetamol. The correct answer (4000) is the combination that unlocks the safe.

- The safe contains a doctor’s report on patient Mad Berger. The report indicates that he is a psychiatric patient, diagnosed with a paranoid personality disorder and paranoid schizophrenia. The report points out that he is apparently obsessed by the thought of being betrayed by his ex-girlfriend (Mrs. Keller) and her new husband, his old lab partner and aims to take revenge.

### Minutes 65-75: Showdown

The countdown ends and the intern calls the students to the main space, where they compile the information. With correct interpretation of clues, the students have found the right solution for the tasks on the whiteboard:

1) The pathogen is *Listeria* monocytogenes

2) The right treatment is Ampicillin. Pregnancy has to be considered when choosing medication.

3) The apple juice is contaminated and needs to be removed to stop the disease spread. The health authority has to be notified for tracing attendees of the suspected spreading event.

Dr. House enters the room. The students report their findings to her. She is very relieved and thanks the students for their help. She hurries off to the emergency room to treat Mrs. Keller.

Mad enters the room. He asks the students what they were doing in his lab and wants to pick up his rat and his medication. Apparently confused, he tells the story of how he wanted to poison the unborn child of his ex-girlfriend because of his grief and jealousy about her new family. A psychiatry nurse enters the room and finds Mad. He explains that Mad ran away from the psychiatry ward and he was looking for him. He asks the students if he can take Mad with him and which medication Mad should get in his state. The students can choose from the medicine packages in the laboratory (Quetiapine, Valerian or Haloperidol) and admit Mad to the psychiatry ward. Mad and the nurse leave the room, heading back to psychiatry.

### Minutes 75-80: The End

The intern announces a time leap of around two months: The transmission of the disease was stopped, Mad´s condition has improved, and Mrs. Keller and her baby are doing well. Mrs. Keller enters the room with a basket full of presents (medical textbooks) and thanks each student individually for his or her special skill. The game ends.

Students, actors, team and experts gather to sit down and discuss the escape room.
