# Supplementary material for: Game-based learning in undergraduate medical education: evaluation of an interdisciplinary escape room
Source: BMC Med Educ. 2025 Nov 15;25:1606. doi: 10.1186/s12909-025-07990-2 (PMC12619439; doi:10.1186/s12909-025-07990-2)
Supplement: Supplementary file 4 — Supplementary Material 4. [file 12909_2025_7990_MOESM4_ESM.docx]

**Medical Escape Room**

(Pre-test questionnaire)

Dear Participants,

Thank you for agreeing to participate in our survey for evaluating the escape room teaching unit. Before beginning the escape room, we ask you to complete the following questionnaire. At the end of the escape room, we ask you to complete another questionnaire with additional questions to evaluate the escape room. Please note that participation in this survey is voluntary. The information obtained may be used pseudonymously for research and evaluation purposes.

The aim of this survey is to:

1. evaluate the escape room to find out which aspects can be improved;
2. evaluate the learning outcomes of the teaching methodology;
3. evaluate knowledge transfer; and
4. determine the needs and approaches of the teaching and the trainers.

To keep your data anonymous, please assign a unique code to your questionnaire. The same code should be used for both the pre- and post-questionnaires. For example, you could use your mother’s initials followed by your birth day: **Kiara Schmidt, June 21, 1999 = KS21**

I agree to the evaluation of the questionnaire for scientific purposes: □ Yes □ No

Have you already heard about the contents of the medical escape room from others (fellow students)?

□ Yes □ No

**Section 1**

| Code (Two letters followed by  two numbers): __________________ | Year of birth:  __________ | Semester:  ___________ | Gender:  M \| F \| D |
| --- | --- | --- | --- |
| Medical student: □ Yes □ No  Other: _______________ | Aspired specialty:  ________________________ | | |

**Section 2**

For each question, **one or more** answers may be correct or incorrect. Please assess each answer option individually as to whether it applies or not.

**1. You are a doctor on duty in an overcrowded emergency room and are caring for a 32-year-old patient. The patient, distraught and in tears, reports that her boyfriend has just broken up with her. When you try to take her medical history, she screams, "Leave me alone!" Which response(s) is/are most appropriate for successful doctor-patient communication?**

1. I reply that I have the impression that the anamnesis cannot be continued at the moment
2. I offer to let her rest for a moment and take care of other patients in the meantime
3. I ignore the request and continue the anamnesis
4. I sit in an open attitude towards the patient and convey to her that I have time
5. I leave the room and send another doctor to her

**2. Which question(s) is/are least relevant in a brief infectious disease history?**

1. Travel
2. Duration and type of symptoms
3. Cases of illness in the area
4. Eating habits
5. Family history

**3. Which statement(s) regarding transfer from the edge of the bed to the wheelchair by a caregiver is/are generally true?**

1. If a second assistant holds the wheelchair, the brakes can remain released during the transfer
2. The height of the wheelchair should be adjusted below the edge of the bed
3. The caregiver can position their knee between the legs of the person being transferred for stabilization
4. The person to be transferred can support the transfer by giving instructions and helping
5. The footrests on the wheelchair remain firmly attached to the wheelchair

**4. What protective clothing is indicated if a gastroenterological infectious disease is suspected?**

1. Hood
2. Protective gown
3. Gloves
4. Simple protective mask
5. FFP2 mask

**5. Which criterion(s) for hygienic hand disinfection in healthcare facilities is/are correct according to the recommendation of the Robert Koch Institute?**

1. Nail polish is permitted for hospital staff, unlike artificial nails
2. Jewelry is a risk on the hands as a reservoir of pathogens
3. Hand disinfection is indicated before entering the break room
4. The exposure time of disinfectant is about 15 seconds
5. According to the World Health Organization, a standardized sequence of movements is prescribed for hand disinfection

**6. Which of the following pathogens belongs/belong to the gram-positive rods?**

1. Clostridia
2. Listeria
3. Staphylococci
4. Bacillus species
5. Pseudomonas species

**7. What information(s) must normally be known to ensure reliable patient identification?**

1. Birth date
2. First name
3. Diagnosis
4. Gender
5. Case number

**8. Which of the following pathogens is/are notifiable by name in Baden-Württemberg?**

1. SARS-CoV-2
2. Dengue virus
3. Human immunodeficiency virus
4. Listeria
5. Echinococci

**9. Which classification(s) of vulnerable groups to infectious pathogens is/are correct according to the Robert Koch Institute?**

1. Pregnant women and Covid-19
2. Swimmers and Klebsiella
3. Immunosuppressed and Candida Albicans
4. Ventilated patients and Staphylococcus epidermidis
5. Intravenous drug addicts and HIV

**10. What is/are the maximum total daily dose for non-opioid analgesics?**

1. Ibuprofen 1000 mg
2. Acetylsalicylic acid 2000 mg
3. Paracetamol 4000 mg
4. Diclofenac 30 mg
5. Metamizole 3000 mg

**11. Which finding(s) would you classify as a personality disorder with paranoid patterns according to ICD-11?**

1. Disinterest in other people
2. Jealousy mania
3. Externalization of guilt
4. High ego dystonia
5. Lack of empathy

**12. What is this picture* about?**


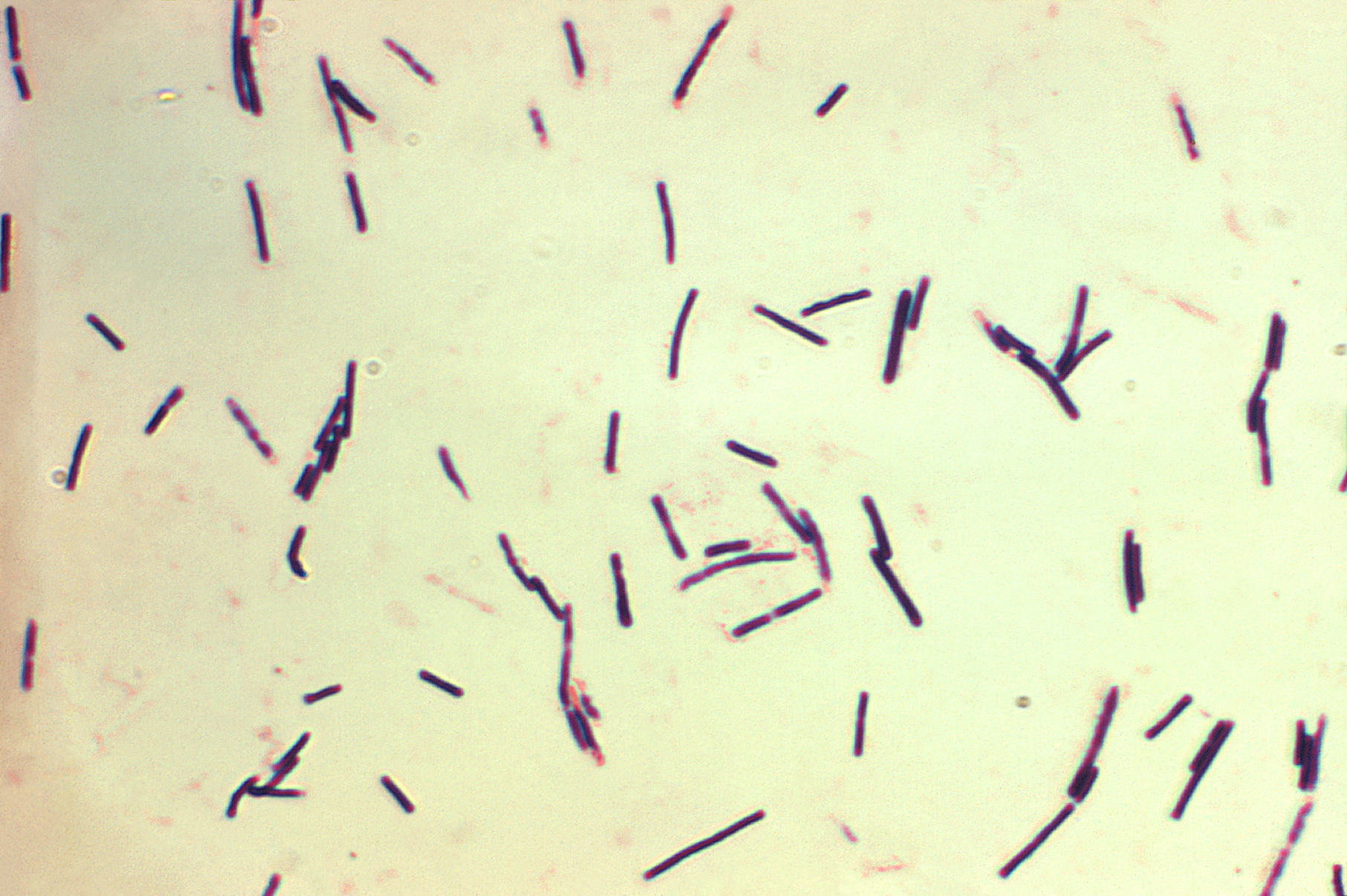


1. gram-positive rods
2. gram-negative rods
3. Bacteria with a thick murein layer
4. Bacteria with a thin murein layer
5. Bacteria that can be easily stained using the Ziehl-Neelsen stain

**13. Which finding(s) in the cerebrospinal fluid clearly indicate bacterial meningitis?**

1. Cloudy texture
2. Lactate 4 mmol/L (reference value 1.5-2.1 mmol/L)
3. Total protein 1200 mg/L (reference value 150-450 mg/L)
4. Cell count 500/µL (reference value <5/µL)
5. Glucose 4.2 mmol/L with serum glucose 5.6 mmol/L (reference value: 2.2-3.9 mmol/L)

**14. Which of the following antibiotics is/are the drug of choice during pregnancy?**

1. Ampicillin
2. Ceftriaxone
3. Flucloxacillin
4. Gentamicin
5. Clarithromycin

**15. Which statement(s) regarding transmission routes of infectious diseases is/are true?**

1. Temperatures of 30°C prevent the spread of Legionella in hot water systems
2. Borrelia can also be transmitted through a bite from wild animals such as foxes
3. To protect against toxoplasma, pregnant women should avoid cleaning litter boxes
4. Listeria can multiply in contaminated products even in the refrigerator
5. Campylobacter can be transmitted directly from person to person

**16. What should be considered for a successful handover in everyday clinical practice?**

1. Record keeping
2. Patient's situation
3. Patient background
4. Current condition of the patient
5. Recommendation for further treatment

**17. A 35-year-old patient with paranoid schizophrenia presents to your psychiatrist's office for the first time. You learn from his medical records that numerous treatment attempts have been made since his diagnosis at age 18, all without success. Which of the following drugs is/are not considered the treatment of choice for treatment-resistant schizophrenia?**

1. Amitriptyline 25 mg/day
2. Haloperidol 1 mg/day
3. Clozapine 400 mg/day
4. Mirtazapine 25 mg/day
5. Quetiapine 50 mg/day

**18. Which situation(s) justify(s) emergency inpatient admission to a psychiatric hospital under guardianship law?**

1. Patient wants to kill partner immediately by knife attack
2. Patient wants to kill partner by poisoning
3. Patient has suicidal intentions with pills
4. Patient wants to harm his-/herself by starving herself
5. Patient does not want to be examined even though amputation of the ring finger is threatened

**19. Which strategy(s) are suitable for improving interdisciplinary team communication?**

1. Communicate therapy changes retrospectively
2. Leave treatment goals open
3. Prevent misunderstandings
4. Promote structured task distribution
5. Strive for a hierarchical team structure

**20. How would you rate your knowledge of the topics covered in the test on a scale of 1-6 (1 = very good, 6 = unsatisfactory)?**

1 2 3 4 5 6

___________________________________________________________________________

**Thank you!**

* License: CDC/Don Stalons - This media comes from the [Public Health Image Library](https://phil.cdc.gov/) (PHIL), with identification number [#2995](https://phil.cdc.gov/details.aspx?pid=2995) from the [Centers for Disease Control and Prevention](https://en.wikipedia.org/wiki/de:Centers_for_Disease_Control_and_Prevention). This image is in the public domain and thus free of any copyright restrictions. Available at: <https://phil.cdc.gov/details.aspx?pid=2995>.

**Medical Escape Room**

(Post-test questionnaire)

Dear Participants,

Thank you for agreeing to participate in our survey for evaluating the escape room teaching unit. Before beginning the escape room, we ask you to complete the following questionnaire. At the end of the escape room, we ask you to complete another questionnaire with additional questions to evaluate the escape room. Please note that participation in this survey is voluntary. The information obtained may be used pseudonymously for research and evaluation purposes.

The aim of this survey is to:

1. evaluate the escape room to find out which aspects can be improved;
2. evaluate the learning outcomes of the teaching methodology;
3. evaluate knowledge transfer; and
4. determine the needs and approaches of the teaching and the trainers.

To keep your data anonymous, please assign a unique code to your questionnaire. The same code should be used for both the pre- and post-questionnaires. For example, you could use your mother’s initials followed by your birth day: **Kiara Schmidt, June 21, 1999 = KS21**

I agree to the evaluation of the questionnaire for scientific purposes: □ Yes □ No

Have you already heard about the contents of the medical escape room from others (fellow students)?

□ Yes □ No

**Section 1**

| Code (Two letters followed by  two numbers): __________________ | Year of birth:  __________ | Semester:  ___________ | Gender:  M \| F \| D |
| --- | --- | --- | --- |
| Medical student: □ Yes □ No  Other: _______________ | Aspired specialty:  ________________________ | | |

**Section 2**

For each question, **one or more** answers may be correct or incorrect. Please assess each answer option individually as to whether it applies or not.

**1. You are a doctor on duty in an overcrowded emergency room and are caring for a 32-year-old patient. The patient, distraught and in tears, reports that her boyfriend has just broken up with her. When you try to take her medical history, she screams, "Leave me alone!" Which response(s) is/are most appropriate for successful doctor-patient communication?**

1. I reply that I have the impression that the anamnesis cannot be continued at the moment
2. I offer to let her rest for a moment and take care of other patients in the meantime
3. I ignore the request and continue the anamnesis
4. I sit in an open attitude towards the patient and convey to her that I have time
5. I leave the room and send another doctor to her

**2. Which question(s) is/are least relevant in a brief infectious disease history?**

1. Travel
2. Duration and type of symptoms
3. Cases of illness in the area
4. Eating habits
5. Family history

**3. Which statement(s) regarding transfer from the edge of the bed to the wheelchair by a caregiver is/are generally true?**

1. If a second assistant holds the wheelchair, the brakes can remain released during the transfer
2. The height of the wheelchair should be adjusted below the edge of the bed
3. The caregiver can position their knee between the legs of the person being transferred for stabilization
4. The person to be transferred can support the transfer by giving instructions and helping
5. The footrests on the wheelchair remain firmly attached to the wheelchair

**4. What protective clothing is indicated if a gastroenterological infectious disease is suspected?**

1. Hood
2. Protective gown
3. Gloves
4. Simple protective mask
5. FFP2 mask

**5. Which criterion(s) for hygienic hand disinfection in healthcare facilities is/are correct according to the recommendation of the Robert Koch Institute?**

1. Nail polish is permitted for hospital staff, unlike artificial nails
2. Jewelry is a risk on the hands as a reservoir of pathogens
3. Hand disinfection is indicated before entering the break room
4. The exposure time of disinfectant is about 15 seconds
5. According to the World Health Organization, a standardized sequence of movements is prescribed for hand disinfection

**6. Which of the following pathogens belongs/belong to the gram-positive rods?**

1. Clostridia
2. Listeria
3. Staphylococci
4. Bacillus species
5. Pseudomonas species

**7. What information(s) must normally be known to ensure reliable patient identification?**

1. Birth date
2. First name
3. Diagnosis
4. Gender
5. Case number

**8. Which of the following pathogens is/are notifiable by name in Baden-Württemberg?**

1. SARS-CoV-2
2. Dengue virus
3. Human immunodeficiency virus
4. Listeria
5. Echinococci

**9. Which classification(s) of vulnerable groups to infectious pathogens is/are correct according to the Robert Koch Institute?**

1. Pregnant women and Covid-19
2. Swimmers and Klebsiella
3. Immunosuppressed and Candida Albicans
4. Ventilated patients and Staphylococcus epidermidis
5. Intravenous drug addicts and HIV

**10. What is/are the maximum total daily dose for non-opioid analgesics?**

1. Ibuprofen 1000 mg
2. Acetylsalicylic acid 2000 mg
3. Paracetamol 4000 mg
4. Diclofenac 30 mg
5. Metamizole 3000 mg

**11. Which finding(s) would you classify as a personality disorder with paranoid patterns according to ICD-11?**

1. Disinterest in other people
2. Jealousy mania
3. Externalization of guilt
4. High ego dystonia
5. Lack of empathy

**12. What is this picture* about?**


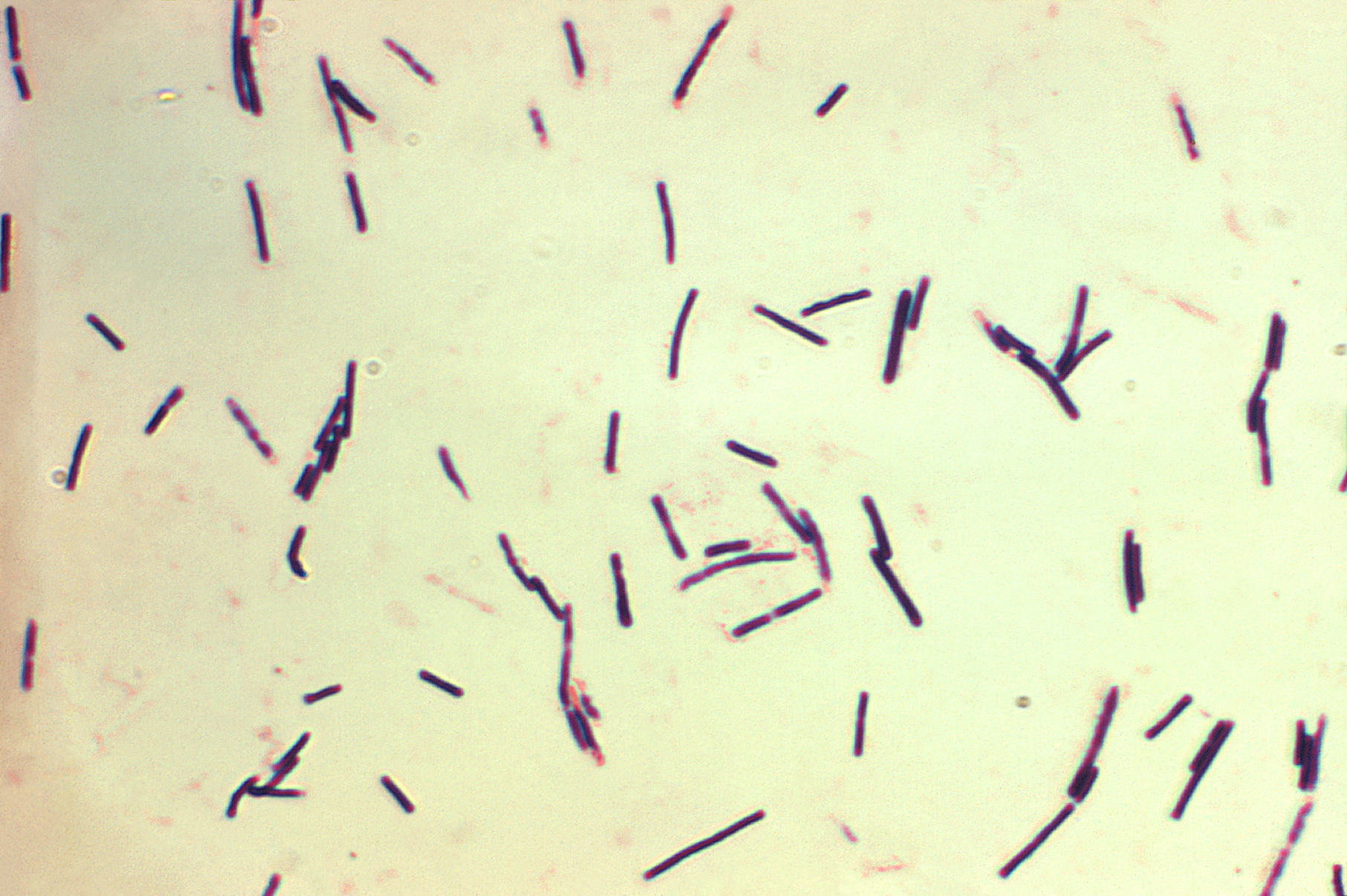


1. gram-positive rods
2. gram-negative rods
3. Bacteria with a thick murein layer
4. Bacteria with a thin murein layer
5. Bacteria that can be easily stained using the Ziehl-Neelsen stain

**13. Which finding(s) in the cerebrospinal fluid clearly indicate bacterial meningitis?**

1. Cloudy texture
2. Lactate 4 mmol/L (reference value 1.5-2.1 mmol/L)
3. Total protein 1200 mg/L (reference value 150-450 mg/L)
4. Cell count 500/µL (reference value <5/µL)
5. Glucose 4.2 mmol/L with serum glucose 5.6 mmol/L (reference value: 2.2-3.9 mmol/L)

**14. Which of the following antibiotics is/are the drug of choice during pregnancy?**

1. Ampicillin
2. Ceftriaxone
3. Flucloxacillin
4. Gentamicin
5. Clarithromycin

**15. Which statement(s) regarding transmission routes of infectious diseases is/are true?**

1. Temperatures of 30°C prevent the spread of Legionella in hot water systems
2. Borrelia can also be transmitted through a bite from wild animals such as foxes
3. To protect against toxoplasma, pregnant women should avoid cleaning litter boxes
4. Listeria can multiply in contaminated products even in the refrigerator
5. Campylobacter can be transmitted directly from person to person

**16. What should be considered for a successful handover in everyday clinical practice?**

1. Record keeping
2. Patient's situation
3. Patient background
4. Current condition of the patient
5. Recommendation for further treatment

**17. A 35-year-old patient with paranoid schizophrenia presents to your psychiatrist's office for the first time. You learn from his medical records that numerous treatment attempts have been made since his diagnosis at age 18, all without success. Which of the following drugs is/are not considered the treatment of choice for treatment-resistant schizophrenia?**

1. Amitriptyline 25 mg/day
2. Haloperidol 1 mg/day
3. Clozapine 400 mg/day
4. Mirtazapine 25 mg/day
5. Quetiapine 50 mg/day

**18. Which situation(s) justify(s) emergency inpatient admission to a psychiatric hospital under guardianship law?**

1. Patient wants to kill partner immediately by knife attack
2. Patient wants to kill partner by poisoning
3. Patient has suicidal intentions with pills
4. Patient wants to harm his-/herself by starving herself
5. Patient does not want to be examined even though amputation of the ring finger is threatened

**19. Which strategy(s) are suitable for improving interdisciplinary team communication?**

1. Communicate therapy changes retrospectively
2. Leave treatment goals open
3. Prevent misunderstandings
4. Promote structured task distribution
5. Strive for a hierarchical team structure

**20. How would you rate your knowledge of the topics covered in the test on a scale of 1-6 (1 = very good, 6 = unsatisfactory)?**

1 2 3 4 5 6

___________________________________________________________________________

**Section 3**

Please answer the following questions about the escape room teaching session using a grade (1-6, 1 very good/exactly applies, 6 very bad/does not apply at all).

| 1. | Please rate the session on the whole. | 1 2 3 4 5 6 |
| --- | --- | --- |
| 2. | Overall, I enjoyed the course. | 1 2 3 4 5 6 |
| 3. | The teaching format was suitable for increasing my knowledge. | 1 2 3 4 5 6 |
| 4. | The course increased my interest in the topics covered. | 1 2 3 4 5 6 |
| 5. | The escape room teaching unit motivates to engage with the topics before or after the teaching unit and encourages self-study. | 1 2 3 4 5 6 |
| 6. | The learning atmosphere during the escape room lesson was positive. | 1 2 3 4 5 6 |
| 7. | I think the teaching format is suitable for remembering learning content in the long term. | 1 2 3 4 5 6 |
| 8. | The event helped me identify my weaknesses. | 1 2 3 4 5 6 |
| 9. | The teaching format encouraged the active use of communication skills. | 1 2 3 4 5 6 |
| 10. | The teaching format promoted teamwork (collaboration skills). | 1 2 3 4 5 6 |
| 11. | The teaching format encouraged the use of leadership skills. | 1 2 3 4 5 6 |
| 12. | The teaching format encourages active participation. | 1 2 3 4 5 6 |
| 13. | The topics covered are relevant for prospective doctors. | 1 2 3 4 5 6 |
| 14. | The escape room lesson was challenging and made me think. | 1 2 3 4 5 6 |
| 15. | I would recommend this course to other students. | 1 2 3 4 5 6 |

Would you like to participate in more escape rooms during your studies? □ Yes □ No

If yes, which subjects/topics do you consider particularly suitable for this teaching format?

__________________________________________________________________________

What did you like the most about this escape room teaching session?

___________________________________________________________________________

___________________________________________________________________________

___________________________________________________________________________

What changes would you make to improve the escape room teaching session?

___________________________________________________________________________

___________________________________________________________________________

___________________________________________________________________________

**Thank you!**

* License: CDC/Don Stalons - This media comes from the [Public Health Image Library](https://phil.cdc.gov/) (PHIL), with identification number [#2995](https://phil.cdc.gov/details.aspx?pid=2995) from the [Centers for Disease Control and Prevention](https://en.wikipedia.org/wiki/de:Centers_for_Disease_Control_and_Prevention) . This image is in the public domain and thus free of any copyright restrictions. Available at: <https://phil.cdc.gov/details.aspx?pid=2995>.
